# Exploring the Potential Utility of Pet Dogs With Cancer for Studying Radiation-Induced Immunogenic Cell Death Strategies

**DOI:** 10.3389/fonc.2018.00680

**Published:** 2019-01-15

**Authors:** Timothy M. Fan, Kimberly A. Selting

**Affiliations:** Comparative Oncology Research Laboratory, Department of Veterinary Clinical Medicine, College of Veterinary Medicine, University of Illinois at Urbana-Champaign Urbana, IL, United States

**Keywords:** immunooncology, comparative oncology, abscopal, immunogenic cell death, radiation, canine, metastases

## Abstract

Radiotherapy serves as a foundational pillar for the therapeutic management of diverse solid tumors through the generation of lethal DNA damage and induction of cell death. While the direct cytotoxic effects of radiation therapy remain a cornerstone for cancer management, in the era of immunooncology there is renewed and focused interest in exploiting the indirect bystander activities of radiation, termed abscopal effects. In radioimmunobiologic terms, abscopal effects describe the radiotherapy-induced regression of cancerous lesions distant from the primary site of radiation delivery and rely upon the induction of immunogenic cell death and consequent systemic anticancer immune activation. Despite the promise of radiation therapy for awaking potent anticancer immune responses, the purposeful harnessing of abscopal effects with radiotherapy remain clinically elusive. In part, failure to fully leverage and clinically implement the promise of radiation-induced abscopal effects stems from limitations associated with existing conventional tumor models which inadequately recapitulate the complexity of malignant transformation and the dynamic nature of tumor immune surveillance. To supplement this existing gap in modeling systems, pet dogs diagnosed with solid tumors including melanoma and osteosarcoma, which are both metastatic and immunogenic in nature, could potentially serve as unique resources for exploring the fundamental underpinnings required for maximizing radiation-induced abscopal effects. Given the spontaneous course of cancer development in the context of operative immune mechanisms, pet dogs treated with radiotherapy for metastatic solid tumors might be leveraged as valuable model systems for realizing the science and best clinical practices necessary to generate potent abscopal effects with anti-metastatic immune activities.

## Significance of a Dog Model—Strengths and Limitations

### Strengths

Domesticated dogs are second only to human beings in terms of being afflicted with naturally-occurring and inherited diseases, and the purposeful breeding of companion dogs for specific characteristics has produced lineage-specific homogeneity that mimics human demographics such as race or geographic phenotypes (1–4). Dogs acquire genetic diseases as do humans, and consequently might serve as suitable comparative models for conserved pathologies, including certain types of cancer (5, 6). Given that pet dogs often share the same environment and are exposed to similar carcinogens as people, the natural development and evolution of canine tumors can closely parallel those that afflict human beings and share comparable recurrence and metastases patterns. The compressed lifespans of dogs in comparison with humans, combined with the substantial veterinary healthcare dollars spent on pet dogs exceeding $15 billion annually (7), provide researchers with a robust population of pet dogs available to participate in studies of cancer pathogenesis and the preclinical assessment of investigational therapeutics and medical devices (8–11). Collectively, the shared genetics of specific canine cancers with their human counterparts (12–17), and the high societal value placed upon dogs as companion animals, uniquely and ethically allow pet dogs to serve as potential valuable large animal models for translational cancer research. Particularly, in the era of immuno-oncology, pet dogs might uniquely serve as ideal parallel tumor models, given the development of spontaneous cancers under competent immune surveillance mechanisms which invariably contributes to shaping of cancer cell immunogenicity and the associated immune topography of the tumor microenvironment (18, 19).

### Limitations

While the recognition of comparative oncologic pathology has been existent for over 50 years (20), the establishment of comparative oncology as a health science discipline by the National Cancer Institute's Center for Cancer Research remains relatively nascent, being formalized in 2003. As such, the purposeful inclusion of pet dogs as parallel cancer models for investigational anticancer immunotherapeutic strategies has only recently begun to bear scientific results in support of the potential model value (21), and has not been maximally leveraged by the scientific cancer research committee given the existence of perceived and true barriers (9), which include heterogeneity of study populations and tumor biology, necessity to conduct adequately powered and prospective clinical trials, and limited availability of diagnostic and therapeutic tools for in-depth scientific investigations. For the study of anticancer immune responses, the diversity and number of commercially available and validated reagents for characterizing immune activation in the domestic canine remain limited in comparison to the existent murine and human reagent toolboxes (22, 23). Additionally, the nuances of immune composition and activation responses in canines is less well-annotated compared to traditional inbreed mouse strains (24–26), however, in aggregate there is sufficient data to support the comparative similarities for specific aspect of the immune system between canines and humans (27, 28).

To expedite the translation of novel immune-based strategies to people with metastatic tumor histologies, the evaluation of experimental therapies in the most highly relevant tumor models should be considered. Besides people, domesticated dogs are also large mammals that develop solid tumors spontaneously that are not only metastatic, but also immunogenic and include canine oral malignant melanoma (OMM) and appendicular osteosarcoma (OS) (29, 30). Importantly, studies demonstrate that these 2 specific solid tumors share similar genetic and histologic features as those found in humans (31–35); suggesting that pet dogs might serve as excellent predictive models for guiding the rational development of immune-based strategies in people with comparable tumor histologies (36).

## Ionizing Radiation Therapy

### Radiation Principles and Mechanisms of Cell Death

The biologic responses of cells exposed to radiation traditionally have been categorized into the 5 R's, being Repopulation, Reassortment, Reoxygenation, Repair, and Radiosensitivity. Understandings of these foundational cellular reactions to ionizing radiation have been leveraged to maximize the anticancer activities of radiation therapy (37, 38). The primary target for radiation cellular damage is DNA, and with low linear energy transfer radiation, such as photons and electrons, single strand DNA breaks are created, accumulate, and mimic damage similar to double strand breaks that become difficult, if not impossible, to repair. Consequently, irreparably damaged cells can no longer replicate limitlessly, and the primary cause of cellular death is mitotic catastrophe (39, 40). Irradiated cells can also undergo apoptosis rapidly following radiation exposure with this form of death most relevant to lymphoid cells (39). Other death pathways also play roles in response to radiation, including autophagy and necrosis. Autophagy involves internal degradation of organelles for the promotion of cellular survival and occurs after radiation as a survival mechanism; but can also progress to cellular death and influence inherent radiosensitivity (41, 42). Lastly, by extensive cellular stress through DNA damage, radiation can induce cellular senescence with consequent tumor cell growth arrest (43, 44).

### Radiation-Induced Immunogenic Cell Death and Abscopal Effects

While anticancer activities from radiation have traditionally been ascribed to direct DNA damage to tumor cells, in the era of immunooncology, there has been focused interest to understand the indirect or “out-of-field” immunomodulatory activities induced by radiation therapy. Specifically, a unique form of radiation-induced cell killing called immunogenic cell death (ICD) holds promise for activating systemic immunity against tumor masses distant from the field of radiation delivery (45), a phenomena termed abscopal effect (46). The regressive activity of local irradiation on distant metastatic cells, constituting the abscopal effect, is attributed to an immune-mediated response (47). Given the recognized potential to amplify systemic anticancer immunogenicity following localized radiation, excitement has been garnered by the scientific community to understand and harness the promise of radioimmunotherapy (48, 49).

Mechanistically, ICD has been a focus of radiobiology research and requires activation of the innate immune system through the release of damage-associated molecular patterns (DAMPs) or alarmins, which are released from injured, stressed, or dying cells within the radiation field (50). Scores of different endogenous alarmins derived from cellular organelles and extracellular matrix proteins have been described (51); however, three specific molecules appear to be required for optimal dendritic cell activation and immune priming against malignant cells, specifically being membrane localization of calreticulin and the release of high mobility box group 1 (HMBG1) and adenosine triphosphate into the tumor microenvironment (52). Collectively the expression and secretion of alarmins by dying cells create a localized milieu which exert either “eat me” or “come find me” signals, and are capable of activating innate immune cells exhibiting cognate DAMPs receptors (TLR, RAGE, P2X7), which leads to the priming of cytotoxic T lymphocytes for an adaptive anticancer immune response (53). Given their immune activating properties, the purposeful induction of alarmins within the tumor microenvironment as an *in-situ* vaccine strategy is actively being investigated (54, 55).

While the elicitation of ICD within the primary tumor microenvironment through ionizing radiation has potential to prime the innate immune system, there remains the necessity for generating sufficient out-of-target tumor responses known as the abscopal effect, especially at sites of metastatic burden that might be unamendable to conventional localized treatment strategies. Despite the documentation of abscopal activities induced by localized radiation therapy in combination with adjunctive treatments (cytokines and chemotherapy), the fraction of human cancer patients that reliably demonstrate abscopal activities sufficient to induce macroscopic tumor regression remains <30% (56). The contextual scenarios (tumor type, host environment, therapeutic combinatorial sequencing) by which abscopal effects can be generated by radiation therapy remain incompletely defined (57, 58). As such, prospective investigations with high-value animal models could accelerate the identification of ideal circumstances to augment the proportion of human cancer patients whom might benefit from the life-extending activities of radiation-induced ICD and associated abscopal effects.

### Opportunity to Optimize Radiation-Induced ICD Protocols

While several recent investigations have discussed the optimal dose and timing of radiation therapy relative to immunologic intervention, no single protocol is clearly superior to others, and the impact of dose rate is relatively unexplored. Given the non-uniformity of various therapeutic radiation regimens for the management of diverse solid tumor histologies, a significant research barrier exists for the thorough characterization of contributory radiation variables required for optimal radiation-induced ICD. While recent meta-analysis has been conducted to “standardize” immune activating potential of radiation treatment protocols through the comparison of biologic effective dose in preclinical models (59), there remains a scientific need for additional prospectively-designed studies inclusive of model systems that more faithfully recapitulate the natural progression of cancer development under immune evolutionary pressures. This “gap” in knowledge given the absence of an ideal experimental model system, is underscored by the rarity of achieving radiation-induced abscopal effects in human cancer patients (56, 60–62). As such, the consistent and reproducible generation of clinically meaningful abscopal effects in most cancer patients remains infrequent and suggests that the current state of understanding regarding radiation-induced immune activation remains incomplete and necessitates the inclusion of complementary innovative modeling systems.

One mechanism to generate new knowledge regarding the feasibility and limitations of radiation-induced ICD and associated abscopal effects could include the rational inclusion of pet dogs with solid tumors. Therapeutic management of cancer in pet dogs parallel the same modalities in human cancer patients, with the inclusion of radiation therapy for controlling localized tumor progression and associated morbidity. Importantly, the repertoire of cognate receptors including toll-like receptors responsible for detecting the presence of pathogens (pathogen associate molecular patterns) and danger signals (damage associated molecular patterns) have been recently characterized in the domestic canine (26, 63–65). With existing tools and knowledge of radiobiology and immunology in the canine species, an opportunity exists to prospectively and systemically evaluate novel radiation-induced ICD strategies in pet dogs that could be translated into life-extending abscopal activities in human cancer patients.

## Relevant Solid Tumors in Pet Dogs for Optimizing Radiation Abscopal Effects

### Canine Oral Malignant Melanoma (OMM)

Malignant melanoma is a metastatic solid tumor affecting both dogs and people (66), however, the anatomic locations of primary tumors differ, with oral cavity and skin being the primary sites for malignant melanoma in dogs and humans, respectively. In canines, melanoma is considered the most common oral malignancy, accounting for ~40% of all oral cancers (67). Despite differences in primary anatomic site, prominent molecular drivers of malignancy are conserved between dogs and people, including AKT and MAPK signaling pathways (31).

**Figure 1:**
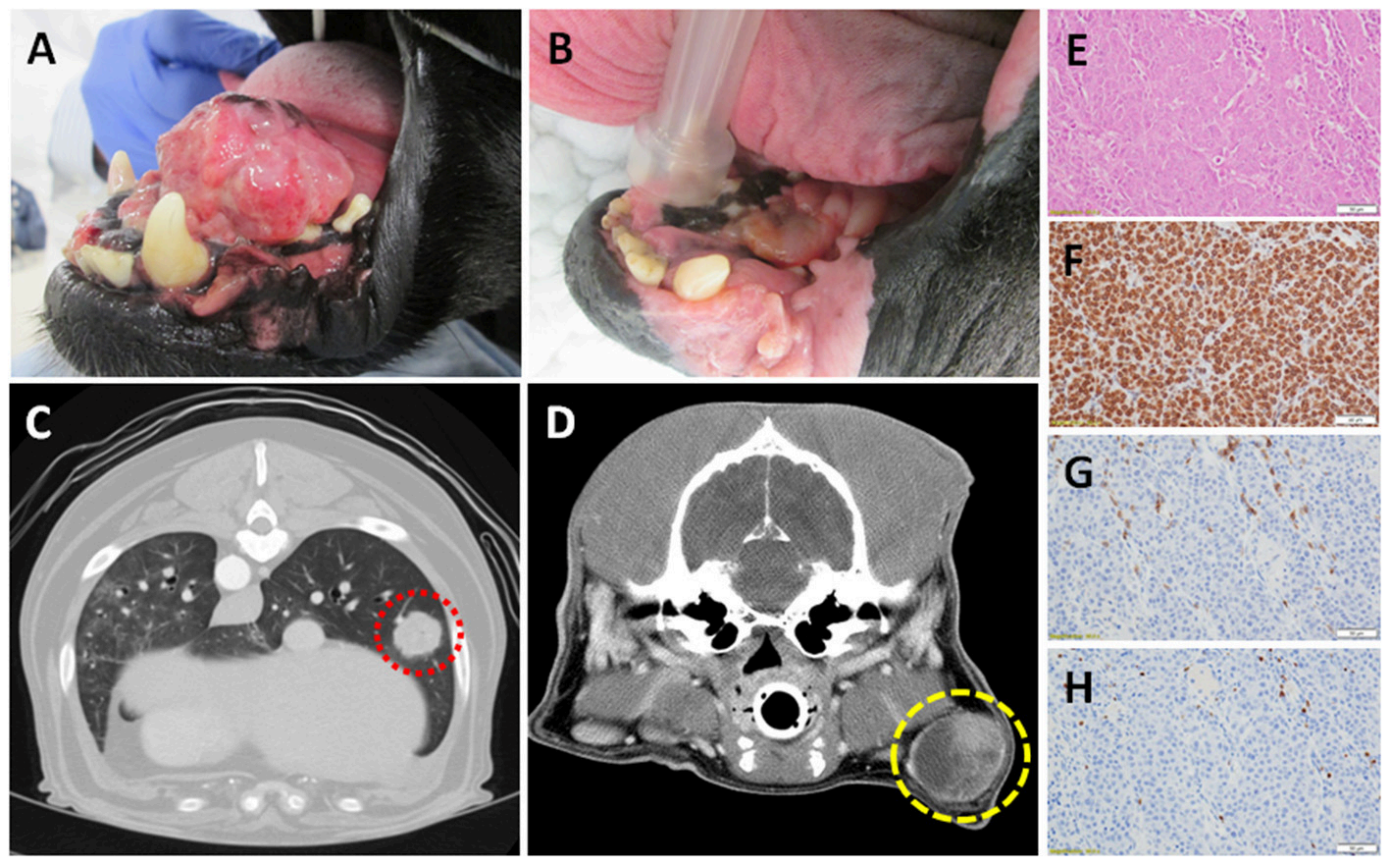
Canine OMM **(A)** pre- and **(B)** post- palliative radiation therapy; note the achievement of strong partial response of primary tumor (courtesy of Dr. Michael Kent, UC Davis). Computed tomography of **(C)** distant pulmonary (red) and **(D)** regional lymph node metastases (yellow) of OMM origin; demonstrating the potential for reproducible and quantitative volumetric assessments for documentation of abscopal activities. Panel (**E–H**; top to bottom) represents the histologic and immunobiologic assessment of a regional lymph node effaced with amelanotic melanoma by H&E, PD-L1, CD3^+^ tumor-infiltrating lymphocytes, and regulatory T cells (courtesy of Dr. Jonathan Samuelson, UIUC). Magnification 400x.

Effective management of canine OMM requires local treatment strategies, as well as systemic intervention to delay the onset and progression of regional and/or distant metastases (68–72). While surgical resection is feasible for some dogs with rostrally-confined primary tumors, most canines are diagnosed with invasive inoperable tumors, and hypofractionated ionizing radiation is instituted for local tumor control (73–75). Radiation therapy, alone or as an adjuvant to marginal resection, can achieve satisfactory local primary tumor control (Figures 1A,B), however a substantive fraction of dogs will develop metastatic progression within 6–9 months of diagnosis (67, 68, 76). While the most common site for OMM metastases are regional lymph nodes (77, 78), progression of distant metastases within the pulmonary parenchyma can become life-limiting in dogs that have achieved durable local disease control (67) (Figures 1C,D), and the institution of adjuvant cytotoxic agents does not definitively yield any survival benefit (74, 79). As such, no standard-of-care adjuvant therapy in dogs with metastatic OMM exists and creates a unique and ethical opportunity to model novel immunotherapeutic strategies that might not be otherwise possible in human patients. Importantly, commercial reagents for the assessment of immunobiologic endpoints including tumoral expression of PD-L1, tumor-infiltrating lymphocytes, and regulatory T cells have recently been validated in canine tissues (Figures 1E–H).

#### Clinical Evidence for Canine OMM Immunogenicity

With conservation of certain tumor-associated antigens in both humans and dogs (80–82), canine OMM has been explored as a relevant tumor model in evaluating various immunotherapeutic strategies, in particular tumor vaccine (30). Both autologous and xenogeneic (tyrosinase) vaccines exert measurable anticancer activities in subsets of dogs treated, with objective responses being documented in patients with unsatisfactorily controlled primary tumors, as well as regression of regional and distant metastatic lesions (83–85). In addition to tyrosinase as a therapeutic target, a limited number of investigations have characterized the immunogenic targeting of xenogeneic GP100 and adenoviral CD40L transfection through vaccination strategies; demonstrating immunobiologic activities and clinical benefit in dogs with OMM (Table 1) (86, 87).

**Table 1:** Summary of canine melanoma immunogenic strategies.

**Immunotherapeutic strategy**	**Target**	**Number of animals**	**Immunologic endpoint**	**Clinical benefit**
Xenogeneic melanoma-antigen-enhanced allogenic tumor cell vaccine	Human glycoprotein 100 (hgp100)	34 dogs	PBMC cytotoxicity Neutralizing anti-hgp100 antibody	1 CR 5 PR 6 SD
Local adenovector human CD40L immunogene transfection	Human CD40L	19 dogs	Circulating cytokines (TNFα, IL8, IL10) Neutralizing anti-human adenovirus serotype 5 antibody	5 CR 8 PR 4 SD

In addition to vaccines, checkpoint blockade strategies have been recently described in dogs with OMM. Initial studies identified the upregulation of PD-L1 following INF-γ exposure in immortalized canine melanoma cell lines, as well as, PD-L1 expression in 100% (8/8) of spontaneous canine OMM samples (88). A follow-up confirmatory study similarly identified 90% (36/40) OMM samples to express PD-L1, and importantly demonstrated that tumor-infiltrating lymphocytes, both CD4^+^ and CD8^+^, expressed PD-1 (89). Expressions of PD-L1 by melanoma cells and PD-1 by TILs, support the potential for melanoma cells to induce T-cell exhaustion as an immunoevasive mechanism. To confirm the functional immunosuppressive activities of PD-L1 expressions in canine OMM, an anti-PD-L1 antibody was evaluated in dogs with OMM, with suggestive evidence for survival time prolongation in four dogs with pulmonary metastasis when compared to historical controls (90). Collectively these clinical investigations support the relevancy of canine OMM as a naturally-occurring model system for testing immunotherapeutic combinations inclusive of other immunomodulatory strategies such as radiation-induced ICD and abscopal activities.

### Canine Appendicular Osteosarcoma (OS)

Osteosarcoma (OS) accounts for 85% of all skeletal tumors in the dog with an estimated 10,000 dogs diagnosed each year (33, 91), and is a disease primarily afflicting the appendicular skeleton of large and giant breed dogs (33). Similarly, OS is the most common primary focal skeletal tumor in people, being the third most frequent cause of cancer in adolescents (92). The comparative similarities at genetic, molecular, and clinical levels shared between canine and pediatric OS are robust (12, 13, 33–35, 93–97); evidence that strongly emphasize the potential value for the utilization of canine OS to guide investigations related to pathogenesis and novel therapeutic discovery (98).

The biologic behavior of OS is aggressive, starting within the local bone microenvironment but then involving distant organs because of metastatic progression. Although 15% of dogs and 20% of people present with detectable lung metastases, the development of metastatic foci in the absence of chemotherapy is 90% within 1 year for dogs and 80% within 2 years for people (99, 100). While the institution of chemotherapy for OS patients has tripled the cure rate of people (20 → 65%) and doubled the survival time of dogs (130 → 270 days), no substantive improvement in long-term outcomes has been achieved for either species over the past 2 decades despite the institution of dose intensification strategies (101, 102). Given the current therapeutic ceiling, there is clinical need to explore alternative adjuvant therapies that might improve metastatic disease control.

**Figure 2:**
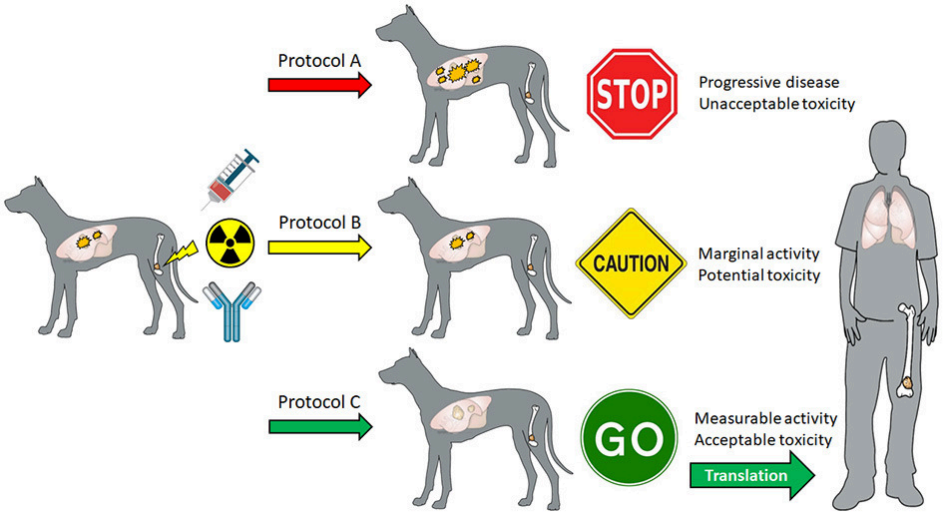
Theoretical schematic for how canine OS patients can be prospectively recruited to evaluate different immunomodulatory strategies inclusive of radiation therapy in combination with other agents such as ICD-inducing anthracyclines (mitoxantrone, doxorubicin, idarubicin), toll-like receptors (CpG ODN, Poly I/C, imiquimod), and checkpoint antibodies (PD-1, PD-L1, OX40) to generate high-value preclinical data to inform “go,” “caution,” or “no go” parallel translational studies maximizing abscopal activities in adolescents diagnosed with OS.

Because the cure rate for canine OS remains <10% 3-years post diagnosis (103), the palliative management of primary tumor malignant osteolysis and associated pain is considered an acceptable treatment option in veterinary medicine (104). Similar to skeletal metastasis in humans, ionizing radiation alone or with bisphosphonates is considered effective for attenuating pathologic bone resorption and associated pain syndromes in affected dogs (105–111), and provides a durable therapeutic window of acceptable analgesia lasting from 3 to 12 months, whereby it is possible to serially monitor for the development, progression, or regression of distant pulmonary metastases. Prospective assessment of combinatorial strategies inclusive of radiation and other immunostimulatory therapies to amplify tumoral lymphocyte infiltrates such as ICD-inducing anthracyclines, toll-like receptor agonists, and checkpoint blocking antibodies which maximally generate robust abscopal effects could be leveraged to guide translational studies in human patients (Figure 2).

#### Clinical Evidence for Canine OS Immunogenicity

Scientific and clinical evidence supports OS to be immunogenic in dogs and humans (29, 112), and strategies that amplify anticancer immunity would be expected to improve long-term outcomes. In dogs, investigations have demonstrated immune activation as an effective strategy for either regressing macroscopic metastases or delaying micrometastatic disease progression. For macroscopic disease, inhalation therapy with liposome interleukin-2 demonstrated the capacity to activate immune cells with consequent regression of measurable pulmonary metastases (113, 114). In the setting of microscopic disease, dogs that develop post-operative wound infection after limb-spare surgery experience prolongation to pulmonary metastases development, with survival times being doubled in dogs that develop osteomyelitis (115, 116), and mechanistically localized infectious inflammation has been linked to NK cell and macrophage activation with consequent mediation of systemic anticancer effects (117). Similarly, L-MTP-PE, a synthetic lipophilic glycopeptide capable of activating monocytes and macrophages to a tumoricidal state, when administered to dogs with OS increases survival time, and underscores the key participation of innate immune cell activation for curbing metastatic progression (118, 119). Lastly, intravenous delivery of a genetically modified *Listeria monocytogenes* to OS-bearing dogs exerts promising anticancer immune activities and extends survival times (120). Collectively, these clinical investigations support the feasibility of stimulating immune effector cells to regress macroscopic and microscopic metastatic disease burdens in dogs diagnosed with OS.

### Emerging Abscopal Modeling in Canine OMM and OS

While existing aggregate data for validating radiation-induced ICD and abscopal activities in pet dogs with cancer remains limited, experimental data is emerging to support the prospective evaluation of hypofractionated radiation therapy for augmenting immune responses. Recently, combinatorial strategies inclusive of ionizing radiation, hyperthermia, and intratumorally delivered virus-like nanoparticle-based therapies have been evaluated in canine OMM, and demonstrate the capacity to elicit immunogenic changes within the localized tumor microenvironment including the promotion TILs into the primary tumor (121, 122). In another investigation conducted in dogs with OMM, abscopal effects were documented in dogs treated with a combination of localized radiation therapy, intratumoral CpG ODN, and an indolamine-2,3-dioxygenase inhibitor (123). For canine OS, combining radiation and immunotherapy has been recently explored in a first-in-dog trial of autologous natural killer (NK) cells (124). In this study, OS-bearing dogs were treated with a coarsely fractionated radiation protocol consisting of 9 Gy once weekly for 4 treatments, with NK cells being harvested and expanded, and then delivered back to dogs by intratumoral injection following the completion of radiation therapy. Of the 10 dogs treated, 5 remained metastasis-free at 6 months, and one had regression of a suspicious pulmonary nodule detected at the time of diagnosis.

## Future Directions and Conclusions

Dogs diagnosed with naturally-occurring cancers of comparative relevance can serve as biology-rich models of disease. If leveraged appropriately, the inclusion of pet dogs can accelerate the discovery of optimal combinations of radiation and immunotherapies which robustly and consistently elicit life-extending abscopal effects. With the availability of linear accelerator-based radiation facilities in veterinary centers analogous to human hospitals, coupled with the development of dog-specific immune-based therapies including vaccines, monoclonal antibodies, and CAR-T technologies, the purposeful inclusion of pet dogs with immunogenic tumors should be seriously contemplated as a unique strategy to aid in defining the limits and benefits of radiation-induced abscopal activities.

The scientific development and clinical assessment of novel immunotherapeutic strategies are rapidly growing areas in veterinary medicine and have demonstrated promise in the settings of canine OMM and OS. Given the conserved biology of these two immunogenic solid tumors between dogs and people, unique opportunities exist collectively for human and veterinary researchers to pilot and validate innovative immune strategies inclusive of radiation therapy in efforts to harness the promise of abscopal anticancer activities.

## Author Contributions

TF and KS project conception and manuscript authorship.

### Conflict of Interest Statement

The authors declare that the research was conducted in the absence of any commercial or financial relationships that could be construed as a potential conflict of interest.
